# Self-treatment of freezing of gait in Parkinson’s disease patients using silicone pads to apply Thai acupressure to plantar acupoints: A randomised, controlled trial

**DOI:** 10.1016/j.prdoa.2024.100254

**Published:** 2024-05-10

**Authors:** Yuka Miyahara, Pattamon Panyakaew, Jiradon Tinuan, Onanong Phokaewvarangkul, Chanawat Anan, Haruki Toriumi, Roongroj Bhidayasiri

**Affiliations:** a Doctor of Philosophy Program in Medical Sciences (International Program), Faculty of Medicine, Chulalongkorn University, Bangkok 10330, Thailand; b Chulalongkorn Centre of Excellence for Parkinson’s Disease & Related Disorders, Department of Medicine, Faculty of Medicine, Chulalongkorn University, and King Chulalongkorn Memorial Hospital, Thai Red Cross Society, Bangkok 10330, Thailand; c Wat Pho Thai Traditional Medical School, Bangkok 10200, Thailand; d Doctor of Philosophy Program in Sports and Exercise Science (Exercise Physiology), Faculty of Sports Science, Chulalongkorn University, Bangkok 10330, Thailand; e Department of Health Development, Faculty of Liberal Arts, Maejo University, Chiangmai 50290, Thailand; f Department of Acupuncture, Shonan Keiiku Hospital, Fujisawa 252-0816, Japan; g Toriumi Acupuncture Clinic, Tokyo 179-0074, Japan; h The Academy of Science, The Royal Society of Thailand, Bangkok 10330, Thailand

**Keywords:** Parkinson’s disease, Acupressure, Quality of life, Kinesthesis, Neurocognitive function

## Abstract

**Introduction:**

Freezing of gait (FOG) involves dysfunction of the motor and sensory systems. Peripheral sensory stimuli, including Thai acupressure, can improve proprioceptive function and decrease FOG episodes. Here, we sought to determine the efficacy of acupressure as a self-treatment to alleviate FOG in patients with Parkinson’s disease (PD).

**Methods:**

We conducted an open-label, controlled trial of 60 PD patients with FOG while medicated, randomised into two groups: an active-treatment group using silicone pads to apply pressure to plantar acupoints on the head of the big toe and the base of the first metatarsal bone on each foot for 6 s using patient body weight while seated, repeated four times for each acupoint bilaterally, and a sham-treatment group using a similar protocol without the silicone pads. The primary outcome was stride length. Secondary outcomes included FOG episodes, FOG duration, percent duration of FOG to total gait time (%FOG), and gait parameters. A baseline-adjusted analysis of covariance was used to compare outcomes between the two groups.

**Results:**

Compared with the sham treatment, the active treatment increased stride length, gait velocity, and cadence (all *p* < 0.001), and decreased FOG episodes and duration (both *p* < 0.001), %FOG (*p* = 0.011), and double-support time (*p* < 0.001). No adverse effects were noted.

**Conclusions:**

Acupressure using silicone pads to stimulate plantar acupoints for self-treatment is a noninvasive, simple, safe way to improve gait and alleviate FOG in patients with PD.

**Clinical Trial Registration:**

We registered the study prospectively in the Thai Clinical Trial Registry No. TCTR20200317001.

## Highlights

• FOG in PD could be attributed to impaired peripheral sensory systems.
• Self-acupressure with silicone pads can alleviate ON-FOG and increased stride length.
• Self-acupressure improved FOG via enhancing proprioceptive feedback.
• An individually determined threshold level of acupressure effectively improved gait and alleviated ON-FOG.

## 1 Introduction

Freezing of gait (FOG), a disabling motor symptom of Parkinson’s disease (PD), is “a brief, episodic absence or marked reduction in the forward progression of the feet despite the intention to walk,” which can result in falls and reduced quality of life [1]. Treatment of FOG is particularly challenging because FOG is often resistant to medication and still occurs (ON-FOG) while patients are medicated to treat parkinsonism (ON-state) [2], [3]. Although the underlying mechanism for FOG remains controversial, it is related to multiple neurotransmitter deficits, including dopaminergic denervation or dysfunction of sensorimotor integration [3], [4], [5]. Sensory deficits play a critical role in contributing to FOG, particularly impaired proprioceptive feedback and abnormal sensory transmission in the peripheral and central nervous systems [4], [6]. Insufficient proprioceptive feedback can disturb kinesthesis and spatial perception and disrupt motor planning, resulting in reduced stride length and FOG [4], [7], [8]. Patients with PD exhibit notable denervation of cutaneous Aβ fibers, including mechanoreceptors [9]. Stimulus of mechanoreceptors in the foot that have the highest sensitivity thresholds in the plantar region for vibratory and touch pressure [10], [11] and modulate somatosensory processing from the plantar nerves [12] might effectively alleviate FOG [13], [14], [15], [16]. This pressure stimulus can be achieved using metallic mechanical stimulators [11], [12], [13], [14] or silicone pads [15], [16] applied to the plantar mechanoreceptors to increase peripheral afferent sensory input and proprioceptive feedback from the plantar nerves to central pattern generators and enhance sensorimotor function, which may improve gait parameters and alleviate FOG.

Acupressure stimulates cutaneous mechanoreceptor sensory afferents, reduces muscle tension and neuromuscular excitability, and improves proprioceptive feedback [17], [18]. Therapeutic Thai acupressure, applied to acupoints at the head of the big toe and the base of the first metatarsal bone, improves gait and alleviates FOG with an effect comparable to that of visual cueing [17]. For patients with PD, the effects of sensory modulating treatments to control gait and balance are merely transient [8]. Rather than in specialised clinics, more frequent stimulation of therapeutic acupoints might be easily achieved by self-treatment outside the clinic using silicone pads to apply pressure to plantar acupoints. However, the efficacy of this self-treatment for alleviating FOG in patients with PD has remained unclear. Here, we sought to determine the efficacy of self-treatment using silicone pads to stimulate plantar acupoints according to the principles of Thai acupressure for improving gait parameters and reducing FOG episodes and their duration.

## 2 Methods

### 2.1 Study design and participants

**Fig. 1:**
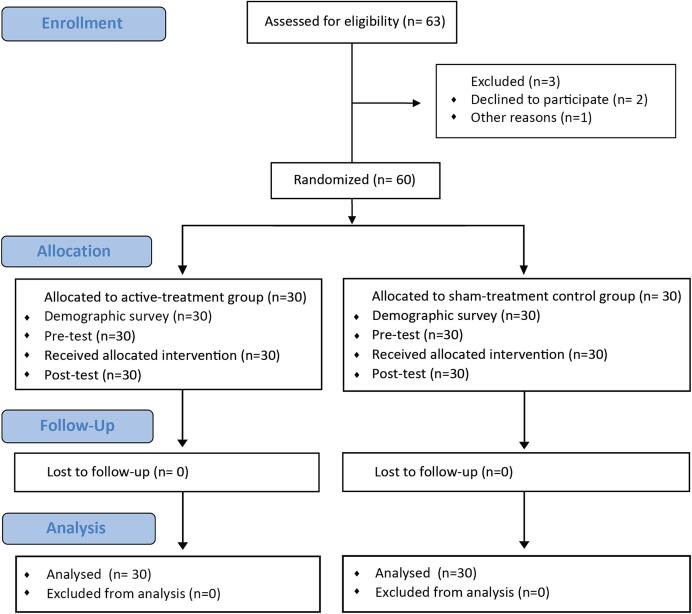
CONSORT randomization flow diagram.

We conducted a randomised, open-label, two-armed, parallel-group, controlled trial. All PD patients were enrolled from the outpatient clinic of the Chulalongkorn Centre of Excellence on Parkinson’s Disease and Related Disorders (ChulaPD, https://www.chulapd.org) at King Chulalongkorn Memorial Hospital, a 1500-bed, tertiary referral, university teaching hospital in Bangkok, Thailand, and were diagnosed according to the United Kingdom Parkinson’s Disease Society Brain Bank criteria. The inclusion criteria were those patients who experienced regular FOG episodes, as defined by scores > 1 for the FOG-questionnaire (FOG-Q) item No. 3 (i.e., “Do you feel that your feet get glued to the floor while walking, making a turn or when trying to initiate walking (freezing)?”) [19], but could walk 10 m independently, had experienced FOG symptoms while taking medication consistently for ≥ 3 months (ON-FOG), had stable anti-PD medication for ≥ 3 months (ON state), and had no cognitive impairment, as determined by item No. 1 of the Unified Parkinson’s Disease Rating Scale (UPDRS) Part Ⅰ with a score ≤ 1 [20]. The exclusion criteria included inability to walk without any assistance, use of deep brain stimulation, had other neurological disorders other than PD, acute visual impairment, severe depression, diabetes-induced peripheral neuropathy, active foot skin conditions, or systolic blood pressure ≥ 140 mmHg and diastolic pressure ≥ 90 mmHg. The protocols used in the present study were approved by the Institutional Review Board (IRB) of the Faculty of Medicine, Chulalongkorn University (IRB No. 211/62). They were conducted according to the principles of the Declaration of Helsinki (1964) and its contemporary amendments (2013), The Belmont Report, CIOMS Guidelines, the International Conference on Harmonization in Good Clinical Practice (ICH-GCP), and registered prospectively with the Thai Clinical Trials Registry (TCTR20200317001). All patients provided written informed consent to participate in the study, which was documented before any intervention. A flow chart of the study enrollment and allocation to each group is shown in Fig. 1.

### 2.2 Protocols

Block randomisation was used to allocate the participants evenly into two groups: an active-treatment group with silicone pads and a sham-treatment group without the silicone pads, and with flat surface. The participants underwent a single session, including a preintervention gait measurement, the allocated intervention, and gait measurement immediately after the intervention on the same day during ON-state, 30-60 minutes after patients took their usual dopaminergic medications.

For baseline characteristics, the Hoehn and Yahr stage, UPDRS-Part III score [20], levodopa equivalent dosage [21], and disease duration were recorded. Spatiotemporal gait parameters, including stride length, gait velocity, cadence, and double-support time, were evaluated before and after the intervention using GAITRite software (version 3.95; CIR Systems). All participants were instructed to walk in a hallway on the pressure-sensitive walkway mat of a GAITRite system for 10 m at their own pace each time, and the gait assessments were repeated twice with 10 min of rest between each assessment [22].

### 2.3 Intervention

The intervention comprised four parts: (1) 10 min of sitting on a chair while resting, (2) intervention guidance, (3) the intervention, and (4) 10 min of sitting on a chair to rest and for blood pressure measurements as vital sign checks. Parts (1) and (4) were the same in both groups. Parts (2) and (3) were conducted with silicone pads in the active-treatment group, and with flat surfaces and without silicone pads in the sham-treatment group. All participants in both groups practised one set of self-acupressure procedures before the actual interventions.

**Fig. 2:**
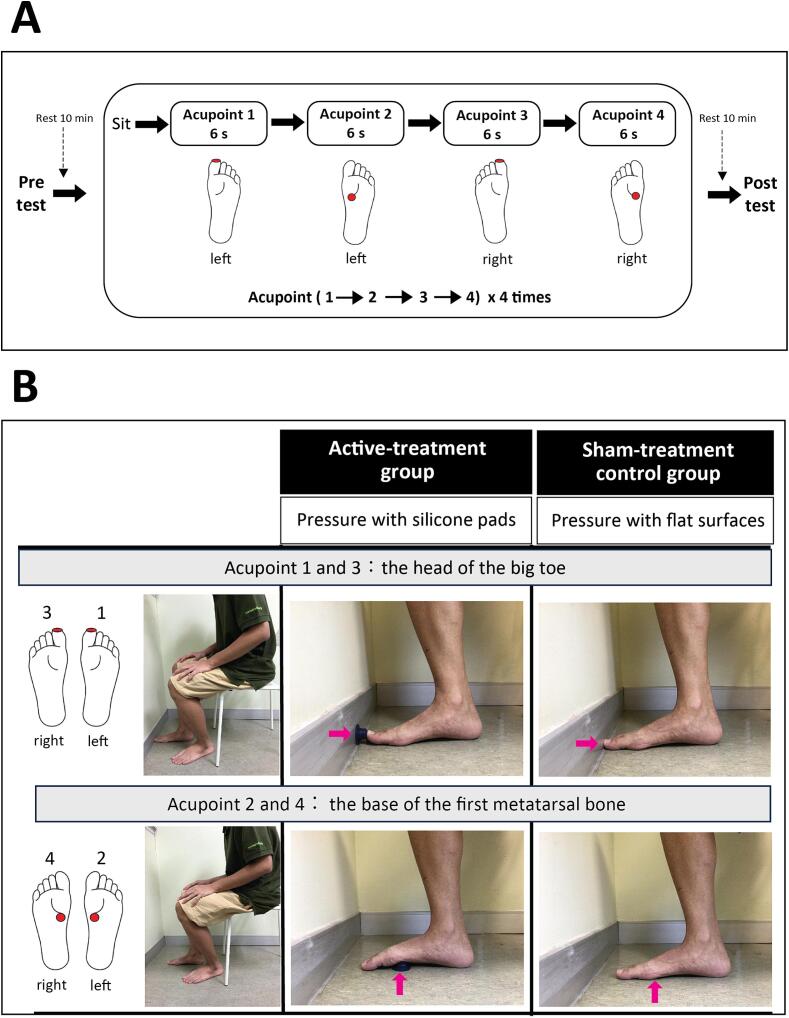
Session flow. The panel A: study design; the panel B: the methods of applying acupressure on the four designated acupoints in active-treatment group and sham-treatment control group.

We observed the patient participants throughout the interventions. The patients could leave if their vital signs were stable after the session (Fig. 2).

#### 2.3.1 Silicone pad acupressure active-treatment group

The participants were instructed to perform self-acupressure by pushing their body weight against the silicone pads at the four designated acupoints separately and in succession: at two points per foot, the head of the big toe and the base of the first metatarsal bone, which influences the monosynaptic reflex in the tibialis anterior muscle, in the active-treatment group [11], [12], [13], [14]. These locations were selected because they have the highest sensitivity threshold for vibratory and touch pressure in patients with PD [10], [11] and have been used in previous studies [11], [12], [13], [14], [15], [16], [17]. Moreover, these locations correspond to standard therapeutic Thai acupoints on the foot that influence motor function in the lower extremities [23]. The participants sat on a chair and were asked to lean their body forward while focusing on their navel at the center of their body to assist in controlling their balance. The intensity of the self-acupressure was gradually increased within 3–5 s until the participants felt mild discomfort, and this individually-determined threshold level of acupressure was sustained for 6 s, and then gradually decreased over approximately 5 s [11], [12], [13], [14]. According to the convention of traditional Thai medicine, acupressure was applied to the left foot first and then to the right foot [23]. The participants were asked to repeat this procedure four times for each acupoint in succession [11], [12], [13], [14] and rest for approximately 5 s between procedures. This stimulus followed previous studies [11], [12], [13], [14] and was coincidentally similar to the process of therapeutic Thai acupressure [17], [18].

#### 2.3.2 Sham-treatment control group

The participants were asked to simply lean their body weight forward when seated, with their foot and the base of first metatarsal bone placed on a flat floor without silicone pads, and the head of their big toe abutting a flat wall. Participants also focused their attention on the acupoints and their navels, as done similarly in the active-treatment group. This protocol was performed with four sets of 3–5 s increasing pressure, maintaining the pressure for 6 s, and 3–5 s while reducing pressure, with an approximately 5 s rest between procedures.

### 2.4 Outcomes

Spatiotemporal gait parameters were measured before and immediately after the intervention in each participant. The primary outcome was stride length. The secondary outcomes centred on the evaluation of FOG, including the number of FOG episodes, FOG duration, the ratio of the total duration of FOG to the total gait duration as a percentage (%FOG) [15], gait velocity, cadence, double-support time, and the coefficients of variation (CV, which represent gait rhythm variation) of stride length, velocity, double-support time, and stride velocity. To be consistent with a previous study, we defined FOG episodes as those with a double-support time ≥ 1.65 standard deviations above the mean and velocity ≤ 90 % below the mean [17], [24]. FOG duration was evaluated objectively based on the footprint results of the GAITRite. The %FOG can indicate the severity of FOG [25].

### 2.5 Statistical analyses

We determined the sample size using a standard formula for a randomised, controlled trial for two parallel groups [26]. We required 52 participants to obtain an 80 % chance of detecting significance at the 5 % level, and we increased the number of participants by 15 % to compensate for possible dropouts. Therefore, the total sample size was 60 participants. Baseline characteristics are summarised using means and standard deviations, or percentages, as applicable (Table 1). For the primary and secondary outcomes, the effects of the silicone pad stimulus at the acupressure points were assessed by comparing the postintervention gait parameters between the active- and sham-treatment groups using an analysis of covariance (ANCOVA) and adjusting the preintervention gait parameters as covariates. A two-sided paired *t* test was used in the post hoc analysis to determine differences in the gait parameters before and after intervention within the treatment groups. We considered differences with *p* < 0.05 to be significant. All the statistical analyses were conducted using IBM SPSS Statistics for Windows (version 22.0).

**Table 1:** Baseline characteristics.

	SAP (n = 30)	Control (n = 30)	Total (n = 60)
**Demographic characteristics**			
Gender (male)	15 (50 %)	15 (50 %)	30 (50 %)
Age (year)	68.93 (7.28)	66.03 (9.05)	67.48 (8.27)
Height (cm)	159.07 (8.64)	159.27 (8.31)	159.17 (8.41)
Weight (kg)	56.27 (10.49)	53.63 (9.38)	54.95 (9.56)
BMI (kg/m^2^)	22.08 (2.70)	21.06 (2.82)	21.57 (2.79)

**Disease-related characteristics**			
PD duration (year)	10.76 (5.48)	9.67 (4.57)	10.20 (5.03)
LED (mg)	886.40 (411.38)	798.43 (487.79)	842.42 (449.56)
HY: ON-state (point)	2.50 (0.51)	2.53 (0.51)	2.52 (0.50)
UPDRSⅢ: ON-state (point)	18.63 (5.48)	19.33 (7.00)	18.98 (6.24)
FOG-Q: ON-state (point)	9.87 (3.41)	9.23 (3.32)	9.55 (3.35)

Data are presented as mean (SD) or n (%) or median (range). SAP, silicone pad acupressure active-treatment group; Control, sham-treatment control group; HY, Hoehn and Yahr stage; BMI, Body mass index; UPDRSⅢ, motor section of the Unified Parkinson’s Disease Rating Scale; LED, levodopa equivalent dosage; FOG-Q, freezing of gait questionnaire; ON-state, period during which the medication has taken effect.

## 3 Results

### 3.1 Participant characteristics

Half of the participants were women. The mean UPDRS-Part III score was 18.98 (6.24) points, the mean PD duration was 10.20 (5.03) years, the mean Hoehn and Yahr stage was 2.52 (0.50), and the mean FOG-Q score was 9.55 (3.35) points, indicating that the participants were in the moderate stages of gait and balance dysfunction (Table 1).

### 3.2 Primary outcome

**Fig. 3:**
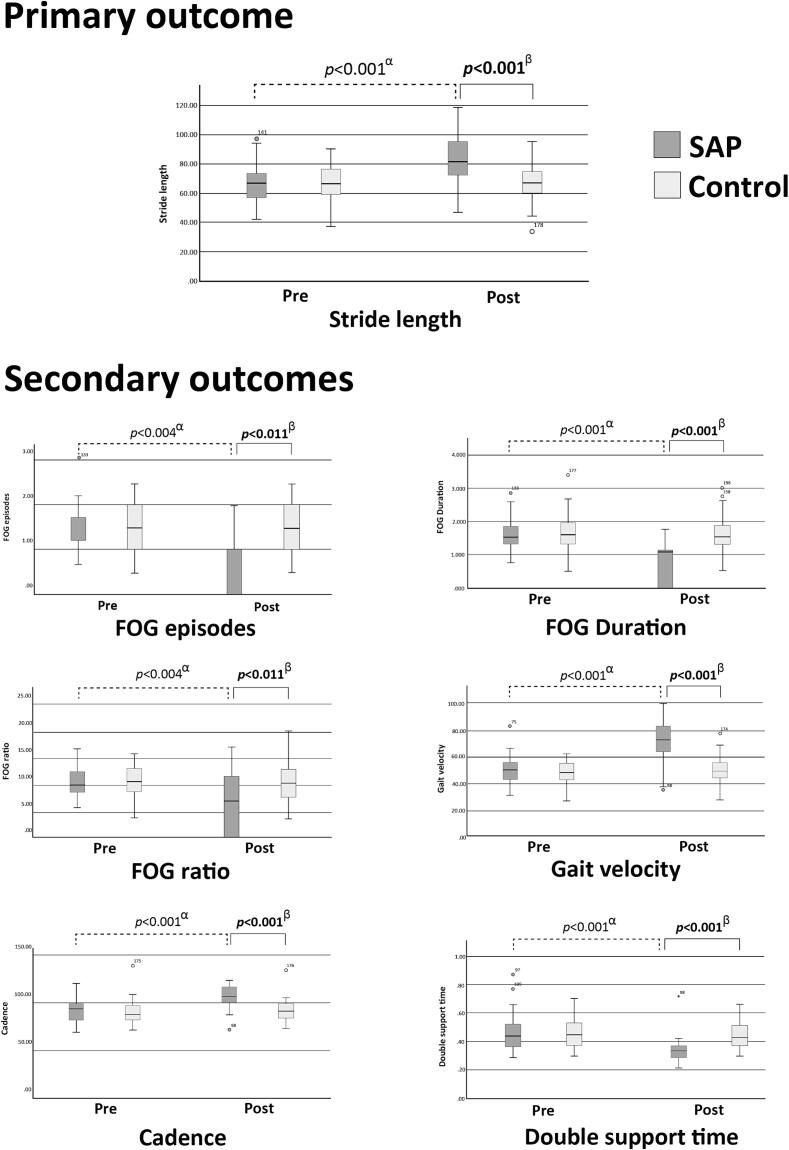
Comparison of the outcomes within and between groups. α: paired *t*-test to compare pre- and post-test within group; β: an analysis of covariance (ANCOVA) test adjusting the preintervention gait parameters as covariates to compare the postintervention gait parameters between the active- and sham-treatment groups. SAP, silicone pad acupressure active-treatment group; Control, sham-treatment control group; FOG, freezing of gait. Statistical significance at *p*-value of < 0.05 (two-sided).

Stride length was significantly greater in the active-treatment group than in the sham-treatment group (95 % CI 13.53; 10.00 to 17.05; *p* < 0.001). According to the within-group analysis, stride length increased significantly in the active-treatment group (*p* < 0.001) after the intervention but not in the sham-treatment group (*p* = 0.138) (Fig. 3, Supplementary Table 1).

### 3.3 Secondary outcomes

After treatment, patients in the active-treatment group showed significant reductions in the number of FOG episodes (95 % CI –0.69; –0.99 to –0.40; *p* < 0.001), FOG duration (95 % CI –0.88; –1.17 to –0.59; *p* < 0.001), %FOG (95 % CI –3.34; –5.87 to –0.80; *p* = 0.011), and double-support time (95 % CI –0.11; –0.14 to –0.08; *p* < 0.001), and increased gait velocity (95 % CI 19.05; 14.48 to 23.62; *p* < 0.001) and cadence (95 % CI 13.23; 8.80 to 17.65; *p* < 0.001) compared with those in the sham-treatment group after treatment. However, there were no significant differences in the CV of stride length, gait velocity, double-support time, or stride velocity. In the within-group analysis, only patients in the active-treatment group showed improvement in secondary outcomes after treatment, except for the CV of stride length, double-support time, and stride velocity. No adverse events were noted for any of the participants (Fig. 3, Supplementary Table 1).

## 4 Discussion

The present study demonstrated that self-treatment using acupressure with silicone pads to stimulate designated plantar acupoints could effectively alleviate FOG during ON-state by increasing stride length, gait velocity, and cadence and decreasing the number of FOG episodes, FOG duration, and %FOG, which is consistent with previously reported findings using other mechanical stimuli of the designated plantar regions [11], [13], [14], [15], [16], [17]. The treatment in which patients repeatedly leaned their seated body weight forward to stimulate acupoints at the head of the big toe and the first metatarsal bone, as targeted by silicone pads placed under the plantar surface, was easy for the patients to perform and can be considered an option for self-treatment outside clinical settings for mildly affected PD patients with ON-FOG. Our findings emphasise the importance of sensory feedback in the pathophysiology of FOG during ON-state.

We hypothesised that deep acupressure with silicone pads targeting the acupoint locations with the highest sensitivity threshold for vibratory and touch pressure might improve peripheral inputs from mechanoreceptors in the regions with sensory deficits [10] better than widely distributed pressure in the plantar region. This improvement may result from pressure stimulus at the designated acupoints in the plantar region, which may sensitise Golgi tendon organs and spindle cells in the tibialis anterior muscle and contribute to dorsiflexion, augmenting heel strike when initiating gait, attributed to impaired anticipatory postural adjustment (APA) [4], [12], [13], [14], [15], [16], [17], [27]. Moreover, pressure stimulus at the big toe is also related to the push-off while initiating gait, resulting in alleviated FOG [13]. Enhanced proprioceptive feedback from increased peripheral sensory afferent input to the central pattern generators may restore defective sensorimotor integration [13], [14], [15], [16], [17]. The stimulus of the four designated acupoints may increase resting-state functional connectivity in brain areas related to visuospatial and sensorimotor integration, as well as APA [12]. In addition, acupressure might stimulate the thalamus and influence the cerebellar locomotor region, which is considered a central proprioceptive integrator for sensory afferents [2], [12]. Consequently, increased proprioceptive feedback activates efferent motor signals and facilitates signal transmission to the spinal cord, improving APA's deficient coupling and motor planning. This process can trigger step initiation, increase stride length, gait velocity, and cadence, and alleviate FOG [1], [4]. The improvement sequence by acupressure may also increase muscle strength and enhance movement [28].

The acupoints were selected based on previous studies [10], [11], [12], [13], [14], [15], [16] and our finding that pressure stimulation at these points with therapeutic Thai acupressure [17] could effectively improve FOG by increasing stride length and reducing FOG episodes with an effect not inferior to visual cues in the immediate phase [17]. The duration of self-treatment was similar to the duration for which therapeutic Thai acupressure techniques were applied at each acupoint [17]. While the participants leaned their seated body weight on the designated points on the plantar surface, they were also asked to focus on their navel. This movement with the focus is a form of motor–cognitive training for patients, which may enhance motor planning and improve FOG [29]. Another possible factor was isometric muscle training of the lower limbs while the patients leaned their body weight against the acupoints, consistent with evidence that isometric training can increase stride length and gait velocity [30], linked to FOG [4], [7], [8]. However, we found no significant changes in spatiotemporal gait parameters in the sham-treatment group, suggesting that motor–cognitive training, including isometric muscle training, might not effectively improve FOG in the immediate phase. The immediate improvement in FOG after the self-treatment using silicone pads may result from enhanced proprioceptive feedback by acupressure rather than motor–cognitive training. We could not find a synergistic effect of acupressure and motor–cognitive training factors in the present study, but this warrants further investigation. Repeated self-treatment with a longer duration may provide potential synergistic effects and reduce the CV of each spatiotemporal gait parameter.

The present study has several limitations, including the fact that it was an open-label study, so blinding patients to treatment was not possible. Nevertheless, the sham treatment control intervention protocol was identical to that for the active-treatment group, except that no silicone pads were used. Pressure stimulus from a flat surface without silicone pads in sham-treated controls could also enhance sensory input to the central nervous system [12], [17]. However, the magnitude of sensory enhancement might not be as great as that of acupressure from silicone pads, where the force is concentrated on the acupoints in the region of the pads [12], [17]. Because the pressure stimulation on the silicone pads at the targeted acupoints was created by the patient’s body weight to a perceived mild discomfort, the differences in pressure intensity for each patient could not be controlled. However, the participants were instructed to determine the pressure intensity with their pain thresholds so that an appropriate stimulus was tailored to each patient, following the characteristics of Thai acupressure. Notably, our finding is that this individually adapted pressure intensity as a self-treatment improves gait and alleviates ON-FOG. We only measured the immediate effect of the pressure stimulus using silicone pads in the plantar area; determining the long-term benefit of this method on gait and balance parameters is warranted. The study was conducted at a single centre in Thailand; therefore, further research will be required to encompass diverse populations.

In conclusion, we suggest that plantar acupressure given through silicone pads is a noninvasive, safe, and simple treatment in which patients with PD can conveniently and sustainably self-administer outside of the clinic to improve their gait and alleviate FOG during ON-state.

## 5 Ethics statement

This study was approved by the IRB of the Faculty of Medicine, Chulalongkorn University (IRB No. 211/62) and is registered in the Thai Clinical Trial Registry (TCTR20200317001). All participants provided written informed consent before participation.

## Author contributions

YM, PP, OP and RB designed the research study. YM, JT and CA performed the research. PP, OP, HT and RB provided help and advice on the execution and critique. YM and PP analysed the data. YM and PP wrote the first draft of the manuscript. RB critiqued and finalised the second draft manuscript. All authors contributed to editorial changes in the manuscript. All authors read and approved the final manuscript. All authors have participated sufficiently in the work and agree to be accountable for all aspects.

## CRediT authorship contribution statement

**Yuka Miyahara:** Conceptualization, Data curation, Formal analysis, Methodology, Writing – original draft. **Pattamon Panyakaew:** Conceptualization, Data curation, Formal analysis, Writing – review & editing. **Jiradon Tinuan:** Data curation, Methodology, Validation. **Onanong Phokaewvarangkul:** Conceptualization, Data curation, Formal analysis. **Chanawat Anan:** Data curation, Formal analysis, Methodology. **Haruki Toriumi:** Validation, Writing – review & editing. **Roongroj Bhidayasiri:** Conceptualization, Funding acquisition, Investigation, Methodology, Project administration, Resources, Supervision, Validation, Visualization, Writing – review & editing.

## Declaration of competing interest

The authors declare that they have no known competing financial interests or personal relationships that could have appeared to influence the work reported in this paper.

## Data Availability

Statistical summaries of the data determined by the study are included in the published article, and further details and data sets are available from the corresponding author after deidentification from any patient and upon reasonable request.
