# Supplementary material for: Self-treatment of freezing of gait in Parkinson’s disease patients using silicone pads to apply Thai acupressure to plantar acupoints: A randomised, controlled trial
Source: Clin Park Relat Disord. 2024 May 10;10:100254. doi: 10.1016/j.prdoa.2024.100254 (PMC11109460; doi:10.1016/j.prdoa.2024.100254)
Supplement: Supplementary Data 1 [file mmc1.docx]

**Supplementary table 1.**

**Within- and between-group comparisons.**

| Outcomes | Group | Pre  (Mean± SD) | Post  (Mean± SD) | Within  group  *p*-value | Δ Post-Pre  (95% CI) | Effect size  (η^2^_p_) | Between  group  *p*-value | Post after adjusted baseline between group  (95% CI) |
| --- | --- | --- | --- | --- | --- | --- | --- | --- |
| **Primary outcome** | | | | | | | | |
| Stride length(cm) ^a^ | SAP | 67.29 (13.17) | 81.78 (16.25) | <0.001^*α^ | 14.48 (11.14 to 17.83) | 0.510 | <0.001^*β^ | 13.53 (10.00 to 17.05) |
|  | Control | 66.78 (12.07) | 67.71 (13.06) | 0.138^α^ | 0.94 (-0.32 to 2.19) |  |  |  |
| **Secondary outcomes** | | | | | | | | |
| FOG (episodes) ^b^ | SAP | 1.45 (0.50) | 0.73 (0.57) | <0.001^*α^ | -0.72 (-0.96 to -0.48) | 0.278 | <0.001^*β^ | -0.69 (-0.99 to -0.40) |
|  | Control | 1.48 (0.61) | 1.43 (0.60) | 0.721^α^ | -0.05 (-0.33 to 0.23) |  |  |  |
| FOG duration (s) ^b^ | SAP | 1.61 (0.47) | 0.75 (0.53) | <0.001^*α^ | -0.86 (-1.10 to -0.62) | 0.390 | <0.001^*β^ | -0.88 (-1.17 to -0.59) |
|  | Control | 1.71 (0.60) | 1.62 (0.59) | 0.618^α^ | -0.09 (-0.44 to 0.27) |  |  |  |
| FOG ratio (%) ^b^ | SAP | 10.58 (2.76) | 7.30 (5.65) | 0.004^*α^ | -3.29 (-5.45 to -1.12) | 0.108 | 0.011^*β^ | -3.34 (-5.87 to -0.80) |
|  | Control | 10.79 (2.96) | 10.63 (3.92) | 0.871^α^ | -0.17 (-2.24 to 1.91) |  |  |  |
| Gait velocity(cm/s) ^a^ | SAP | 50.73 (10.66) | 71.43 (15.42) | <0.001^*α^ | 20.70 (16.42 to 24.97) | 0.550 | <0.001^*β^ | 19.05 (14.48 to 23.62) |
|  | Control | 49.19 (8.03) | 50.81 (9.87) | 0.063^α^ | 1.63 (-0.10 to 3.35) |  |  |  |
| Cadence (steps/min) ^a^ | SAP | 91.98 (11.69) | 106.06 (12.54) | <0.001^*α^ | 14.08 (9.69 to 18.47) | 0.386 | <0.001^*β^ | 13.23 (8.80 to 17.65) |
|  | Control | 90.38 (13.28) | 91.70 (12.00) | 0.212^α^ | 1.32 (-0.79 to 3.4) |  |  |  |
| Double support time (s) ^b^ | SAP | 0.47 (0.13) | 0.34 (0.09) | <0.001^*α^ | -0.13 (-0.17 to -0.10) | 0.500 | <0.001^*β^ | -0.11 (-0.14 to -0.08) |
|  | Control | 0.45 (0.09) | 0.44 (0.08) | 0.069^α^ | -0.02 (-0.03 to 0.00) |  |  |  |
| CV of Stride length (%) ^b^ | SAP | 9.04 (2.73) | 9.21 (4.44) | 0.785^α^ | -0.17 (-1.06 to 1.39) | 0.009 | 0.481^β^ | 0.615 (-1.12 to 2.35) |
|  | Control | 9.28 (4.18) | 8.75 (3.64) | 0.447^α^ | -0.53 (-1.94 to 0.88) |  |  |  |
| CV of Gait velocity (%) ^b^ | SAP | 17.54 (6.34) | 14.48 (4.71) | 0.035^*α^ | -3.07 (-5.90 to -0.24) | 0.028 | 0.205^β^ | -3.41 (-8.74 to 1.91) |
|  | Control | 17.65 (7.49) | 17.98 (16.60) | 0.881^α^ | 0.34 (-4.25 to 4.93) |  |  |  |
| CV of Double support time (%) ^b^ | SAP | 13.41 (5.99) | 12.81 (4.46) | 0.620^α^ | -0.60 (-3.04 to 1.84) | 0.004 | 0.631^β^ | -0.863 (-4.44 to 2.72) |
|  | Control | 16.51 (12.35) | 14.54 (9.32) | 0.382^α^ | -1.96 (-6.49 to 2.26) |  |  |  |
| CV of Stride velocity (%) ^b^ | SAP | 11.42 (2.89) | 10.62 (4.22) | 0.313^α^ | -0.79 (-2.37 to 0.79) | 0.000 | 0.995^β^ | 0.005 (-1.74 to 1.75) |
|  | Control | 11.21 (4.49) | 10.54 (2.89) | 0.382^α^ | -0.67 (-2.17 to 0.84) |  |  |  |
| SAP, silicone pad acupressure active-treatment group; Control, sham-treatment control group; FOG, freezing of gait; CV, coefficient of variation; Pre, pre-test; Post, post-test; a, increasing shows the improvement; b, decreasing shows the improvement;α, paired t-test; β, an analysis of covariance (ANCOVA) test adjusting the preintervention gait parameters as covariates to compare the postintervention gait parameters between the active- and sham-treatment groups; *Statistical significance at *p*-value of <0.05 (two-sided).  FOG episodes were fulfilled as those with a double-support time ≥1.65 standard deviations above the mean and velocity ≤90% below the mean. FOG duration time was corresponded to FOG episodes. FOG ratio was calculated as the ratio of total duration of FOG to the total gait duration as a percentage (%FOG). | | | | | | | | |
